# Insights on the Control of Yeast Single-Cell Growth Variability by Members of the Trehalose Phosphate Synthase (TPS) Complex

**DOI:** 10.3389/fcell.2021.607628

**Published:** 2021-01-28

**Authors:** Sevan Arabaciyan, Michael Saint-Antoine, Cathy Maugis-Rabusseau, Jean-Marie François, Abhyudai Singh, Jean-Luc Parrou, Jean-Pascal Capp

**Affiliations:** ^1^TBI, Université de Toulouse, CNRS, INRAE INSA, Toulouse, France; ^2^Electrical and Computer Engineering & Biomedical Engineering, University of Delaware, Newark, DE, United States; ^3^Institut de Mathématiques de Toulouse, UMR5219, Université de Toulouse, CNRS, INSA, Toulouse, France

**Keywords:** stochastic gene expression, *Saccharomyces cerevisiae*, single-cell analysis, gene expression noise, phenotypic heterogeneity, TPS1, TSL1

## Abstract

Single-cell variability of growth is a biological phenomenon that has attracted growing interest in recent years. Important progress has been made in the knowledge of the origin of cell-to-cell heterogeneity of growth, especially in microbial cells. To better understand the origins of such heterogeneity at the single-cell level, we developed a new methodological pipeline that coupled cytometry-based cell sorting with automatized microscopy and image analysis to score the growth rate of thousands of single cells. This allowed investigating the influence of the initial amount of proteins of interest on the subsequent growth of the microcolony. As a preliminary step to validate this experimental setup, we referred to previous findings in yeast where the expression level of Tsl1, a member of the Trehalose Phosphate Synthase (TPS) complex, negatively correlated with cell division rate. We unfortunately could not find any influence of the initial *TSL1* expression level on the growth rate of the microcolonies. We also analyzed the effect of the natural variations of trehalose-6-phosphate synthase (*TPS1*) expression on cell-to-cell growth heterogeneity, but we did not find any correlation. However, due to the already known altered growth of the *tps1*Δ mutants, we tested this strain at the single-cell level on a permissive carbon source. This mutant showed an outstanding lack of reproducibility of growth rate distributions as compared to the wild-type strain, with variable proportions of non-growing cells between cultivations and more heterogeneous microcolonies in terms of individual growth rates. Interestingly, this variable behavior at the single-cell level was reminiscent to the high variability that is also stochastically suffered at the population level when cultivating this *tps1*Δ strain, even when using controlled bioreactors.

## Introduction

The increasing number of studies describing phenotypic heterogeneity in microbial populations, opened a new look at biological phenomena that were thought to be far more homogeneous from cell-to-cell. Genetically identical microbial cells indeed display heterogeneity in their morphology, the composition of their cellular components, and their growth dynamics (Ackermann, 2015). Especially, while decades of research deciphered the molecular mechanisms underlying growth control, particularly upon the availability of key nutrients (Broach, 2012), population-based assays concealed cell-to-cell variations upon growth stimuli or inhibitors such as various stresses (Petrenko et al., 2013).

High-throughput microscopy made it possible to observe and quantify microbial growth heterogeneity at the single-cell level on thousands events (Levy et al., 2012; Ziv et al., 2013, 2017; Kiviet et al., 2014; Van Dijk et al., 2015; Cerulus et al., 2016; Li et al., 2018; Dhar et al., 2019). A pioneering work in *Saccharomyces cerevisiae* demonstrated that growth rate heterogeneity could serve as a bet-hedging mechanism, providing a benefit to the population across changing environments, especially in yeast (Levy et al., 2012). Clonal *S. cerevisiae* populations displayed broad distributions of growth rates with slow growth being predictive of resistance to heat killing in a probabilistic manner (Levy et al., 2012). Cell-to-cell heterogeneity in growth rate was also observed across laboratory strains, natural and clinical isolates, and that independently of differences in population growth rate (Ziv et al., 2013).

Metabolic heterogeneity is acknowledged to be intrinsically linked to growth rate heterogeneity in microbial populations (Takhaveev and Heinemann, 2018; Wehrens et al., 2018). A role for the DNA damage response has also been suggested in the generation and maintenance of proliferation heterogeneity (Van Dijk et al., 2015; Yaakov et al., 2017). Toward the understanding of the molecular and cellular basis for such heterogeneity, it has been shown that the slow-growing subpopulation in *S. cerevisiae* expresses more genes in general (Van Dijk et al., 2015). These results suggested a more permissive chromatin leading to more stochastic and plastic gene expression, which may, in turn, allow cells to explore a larger phenotypic space (Van Dijk et al., 2015). This is detrimental for single cells in terms of growth rate in constant environments, yet advantageous when the cells need to shift to alternative carbon sources, for example, for faster transcriptional reprogramming and shorter lag phases (Venturelli et al., 2015). This phenomenon of pervasive gene expression in a subpopulation is very similar to what was observed in undifferentiated mammalian stem cells that exhibit permissive chromatin allowing widespread and highly variable gene expression (Efroni et al., 2008; Gaspar-Maia et al., 2011), which is associated with a specific metabolic state (Ryall et al., 2015). These data suggested that metabolism, along with stress response and mitochondrial activity, could emerge as a key player in epigenetics, with metabolites used as substrates for chromatin modifiers (Gut and Verdin, 2013).

By looking for genes that first were previously found to be expressed with high noise (Newman et al., 2006) (that could account for their contribution to growth heterogeneity), and second whose deletion strongly affect population growth rate in *S. cerevisiae*, Levy *et al* identified *TSL1*, which encodes a member of the Trehalose Phosphate Synthase (TPS) complex. Using a GFP-tagged version of Tsl1, they revealed that expression of this gene negatively correlated with cell-division rate in microcolonies, with high Tsl1-GFP fluorescence being associated with cells that undergo few or no cell divisions over the first 8 h after spreading (Levy et al., 2012). Interestingly, other genes encoding members of the TPS complex (*TPS1* and *TPS2*), were also identified as potential markers of growth state, without further investigations in their work. The same group then found that different levels of intracellular cyclic AMP (cAMP) between single cells underlid, at least in part, growth rate heterogeneity and stress tolerance, highlighting the importance of cell signaling leading to stress-related transcription factors Msn2 and Msn4 (Li et al., 2018).

These authors also related growth heterogeneity to energetic metabolism when they observed that *CIT1*, encoding citrate synthase that catalyzes the first step in the TCA cycle, was a marker that also correlated with growth rate. Interestingly, *CIT1* expression was negatively correlated with growth rate across all conditions (acetate, glucose, galactose) (Ziv et al., 2013), while a positive correlation was observed within populations in different carbon sources and different glucose concentrations, even if this might be an indirect relationship (Ziv et al., 2013). A recent study that screened the *S. cerevisiae* gene deletion library for the consequences of gene deletion on single-cell variability of growth also found relationships with energetic metabolism. The authors revealed that deletion of mitochondrial functions produced the most important changes in the fraction of slow-growing cells, this phenotypic heterogeneity being especially impacted by variation in mitochondrial membrane potential (Dhar et al., 2019).

Finally, other works found connections between single-cell variability of growth and sugar transport. Cerulus et al. (2016) examined gene expression and single-cell growth on palatinose and showed, by hypothesizing that genes necessary for growth on this sugar might affect the observed growth variability, that overexpressing Mal11 an alpha-glucoside transporter, reduces the division time variability. Similarly, works by Ziv et al. (2017) mapped genetic loci determining variation in lag duration and exponential growth rate using high-throughput microscopy assay in various glucose concentrations, and found that sequence variation in the gene coding for the high-affinity glucose transporter Hxt7 contributes to such variation.

These works revealed a variety of potential pathways and markers that are involved in single-cell variability of growth and that all contribute in part to this complex phenomenon. As mentioned, the candidate molecular markers of slow-dividing cells in *S. cerevisiae* are enriched in genes involved in bioenergetics (Levy et al., 2012), especially those involved in the metabolism of trehalose. Beyond Tsl1 whose expression particularly anti-correlated with individual growth phenotypes (Levy et al., 2012), Tps1 is also of interest because it appears to have alternative functions in metabolism and cell physiology. While this enzyme catalyzes the first step in the trehalose synthesis pathway, converting UDP-glucose and glucose-6-phosphate to trehalose-6-phosphate, it is proposed to control glycolytic flux through trehalose-6-phosphate inhibition of the hexokinase-mediated phosphorylation of glucose at the gate of glycolysis (Blazquez et al., 1993). Thus *TPS1* overexpression could reduce the amount of glucose entering glycolysis and the yeast fermentative capacity (Rossouw et al., 2013). This protein is especially involved in controlling ATP levels (Walther et al., 2013) but also indirectly other metabolites. It was also found to generate equilibrium between two glycolytic states (Van Heerden et al., 2014) and spontaneous, non-genetic variation between cells could create a continuous probability distribution for metabolite concentrations and metabolic fluxes (Van Heerden et al., 2014). Finally, the involvement of Tps1 in many cellular processes led authors proposing that it could be considered as a moonlighting protein (Gancedo et al., 2016). Taken altogether, these data strongly suggest that *TSL1* and *TPS1* have an incidence on growth rate at the population and the single-cell levels through their roles in stress response and metabolism control.

In this work, we developed a new methodological pipeline to gain insight on the putative relationship between yeast single-cell variability of growth and Tsl1 and Tps1 function, especially because variations of the cell metabolic state may originate from *TPS1* expression variability, which has never been studied in this context. We adopted a strategy consisting of first sorting cells depending on high or low expression level of these genes, then scoring the growth rate of thousands microcolonies during the first hours after plating and fixing cells on Concanavalin A (ConA)-coated glass slides. ConA is a plant lectin, enabling fixing yeast α-mannose residues of glycoproteins that are present on the yeast cell wall surface (So and Goldstein, 1968). This strategy allowed us to study growth in a homogenous cell population in terms of size and morphology, and to measure the impact of the initial expression level on the future growth rate. Overall, our results suggested that the initial amount of members of the TPS complex does not influence the future growth rate of the microcolony.

## Materials and Methods

### Strains and Culture Conditions

The tagged yeast strains used in this study derived from the *S. cerevisiae* laboratory auxotrophic strain BY4741 (MATa *his3*Δ*1 leu2*Δ*0 met15*Δ*0 ura3*Δ*0*) (all the strains used in this study are listed in Supplementary Table 1). The *tps1*Δ strain used on microchambered culture slides came from the Open Biosystem's YKO strains collection. The Wild-Type (WT) and *tps1*Δ mutant strains used in batch cultures were previously constructed in the CEN.PK background (Guillou et al., 2004).

For tdTomato tagging, the tdTomato sequence (1514 bp) was amplified by PCR from the plasmid pFA6a-tdTomato-His (team collection) (all the primers used in this study are listed in Supplementary Table 2). For N-terminal fusions (i.e., Ntd-Tsl1 and Ntd-Tps1 strains), we used primers containing 50 bp extensions that overlapped the downstream and upstream sequences of the start codon of the ORF of interest (respectively −50 to −1 and +4 to +53 for Ntd-Tsl1, −50 to −1 and +20 to +69 for Ntd-Tps1). For the C-terminal fusion of Tsl1 (Ctd-Tsl1 strain), the primers were designed with 50 bp extensions overlapping the downstream and upstream sequences of the stop codon. Homologous recombination was facilitated by using the CRISPR-Cas9 strategy. A plasmid derived from pML107 (Addgene) and carrying the gRNA expression cassette inserted at the Sap1 cloning site (sequence in Supplementary Table 2), the Cas9 enzyme sequence and the *LEU2* gene for selection, was transformed in the BY4741 strain together with the tdTomato-amplified sequences. For the Ntd-Tsl1 strain, the Cas9 cleavage site was located directly on the start codon. For the Ntd-Tps1 strain, this cleavage site was located 13 nucleotides downstream the start codon. The PAM sequence and the gRNA expression cassette design were determined with the CRISPR Direct webtool (https://crispr.dbcls.jp/).

For overexpression of *TSL1*, we replaced the native *TSL1* promoter with the promoter of the *S. cerevisiae* gene *TDH3*. Briefly, 767 bp of the *TDH3* core promoter (−767 to −1) were amplified by PCR from the BY4741 genomic DNA, with primers carrying 50 bp extensions mapping on the *TSL1* core promoter sequence of the Ntd-Tsl1 strain (−800 to −760 and +1 to +50). By using the CRISPR-Cas9 strategy and the PAM site used for the construction of the Ntd-Tsl1 strain, this amplified *TDH3* fragment was used to replace 760 bp of the *TSL1* core promoter (−760 to −1), upstream the tdTomato ORF in the Ntd-Tsl1 strain. Increased *TSL1* expression in this new pTDH3-tdTomato-Tsl1 strain was checked by cytometry analysis.

Yeast strains were routinely grown in shake flasks, in YNB medium at 30°C with shaking (250 rpm). YNB medium was composed of 1.71 g/L Yeast Nitrogen Base without amino acids (Euromedex), 0.79 g/L Complete Synthetic Medium (CSM, MP Biomedicals), 5 g/L ammonium sulfate (Sigma). Liquid cultures were supplemented with 20 g/L glucose (Sigma). For experiments including the *tps1*Δ strain, 20 g/L galactose (Sigma) was used as the carbon source, as this strain could not grow on fermentable sugars like glucose. During strain constructions, for the selection of *LEU2* complemented transformants (CRISPR-Cas9 plasmid), plates were prepared with 0.69 g/L CSM-LEU (MP Biomedicals) instead of CSM. Batch cultures of the WT and *tps1*Δ mutant in controlled bioreactors (unpublished results from the team) had been carried out as described in Jules et al. (2005). For a real independency of the replicates, shake-flask pre-cultures and cultures were performed on different weeks.

### Slides Preparation for Microchambered Cultures

For growth assay experiments, microscopy slides (Superfrost Plus, Thermo Scientific) were plasma-treated [0.5 mbar of O_2_, 5 min at 70% generator power (200 w)], in the Plasma O_2_ Pico μW UHP (Diener Electronic) for cleaning and enabling a better coating and spreading of cells. Glass slides were then coated with 200 μL of 1 mg/mL ConA per slide (ConA type VI from *Canavalia ensiformis*, Sigma L7647). These steps were carried out under hood at room temperature and slides were left overnight before being dried for 24 h at 4°C. Prior to use, slides were warmed up at 30°C and adhesive spacers were added (Gene Frame 65 μL, Thermo Scientific AB0577), delimiting a 100 mm^2^ area on the slide. Finally, these devices were rehydrated for ~10 min with 100 μL of medium. This liquid was then replaced by 65 μL of cell suspension, diluted in the appropriate medium to spread ~1,000 single cells / mm^2^, then closed with a coverslip (D263 M, Schott).

### Cell Sorting

The cell sorting experiments were carried out on the MoFlo Astrios EQ cell sorter with the Summit v6.3 software (Beckman Coulter). For pre-cultures, cells were inoculated from stationary phase cells (colony from plate) and were cultivated for 48 h at 30°C with rigorous shaking (200 rpm), with dilutions to 5.10^5^ cells/mL every 12 h to get exponentially growing cells (that is 0.5 to 1.10^6^ cells/mL) at the time when the sorting was performed. The sorting device was set at 30°C. Cell sorting was carried out with 70 μm nozzle and 60 psi operating pressure. The sorting speed was kept around 30,000 events per second. We set the purity mode for the sort mode and 1 drop for the droplet envelope. As is shown in Supplementary Figure 1A, we first selected single cells with similar cell size and granularity based on the FSC-Area vs. SSC-Area and the FSC-Height vs. FSC-Area plots (488 nm laser). Then, based on the histogram of the tdTomato fluorescence (560 nm laser, 614/20 filter), we simultaneously sorted 5% of cells on both sides of the distribution, that is presenting either the highest or the lowest fluorescence intensities (Supplementary Figure 1B), to recover two subpopulations of 50,000 single cells, respectively named “Plus” and “Minus.” To analyze the dynamics of gene expression recovery, 200,000 cells were collected for each “Plus” and “Minus” subpopulation and the fluorescence was followed from 20,000 cells aliquots, for 6 h, by using standard cytometry analysis (see below).

### Cytometry Analysis

To analyze the fluorescence profile of GFP and tdTomato strains, we used the MACSQuant® VYB with the MACSQuantify™ Software (Miltenyi Biotec). A total of 20,000 cells were analyzed per sample, by using the Y2-A Channel (615/20 nm) for tdTomato strains and the B1-A channel (525/50 nm) for the GFP strains. The fsc files were exported and analyzed by the FlowJo software. All the figures were drawn based on log-transformed values of fluorescence intensity.

### Microcolonies Growth Measurement

Microcolony growth analysis was performed on the Morphologi G3-S microscope (Malvern Panalytical). The parameters used for the microscope (x20 magnification, Nikon CFI60 camera, bright field, brightness 70%) and the pre-filtering of the acquired data were the same for all the experiments. A manual pre-filtering was indeed performed directly during image acquisition using the microscope software (Malvern Morphologi v7.21), all the particles with a diameter of <1 μm being considered as background noise and hence excluded from the analysis.

For non-sorted cells, a quick sonication was applied to dissociate clumps before plating (two times 10 s sonication at 20% amplitude). Sorted fluorescent cells did not require this preliminary step and were directly spread on rehydrated slides. In both case, an appropriate dilution was applied to cell suspensions and ~1,000 cells were spread per mm^2^. After 10 min at 30°C, slides were cautiously rinsed once with synthetic medium to wash-out unfixed cells. From our observations, about 10% of the spread cells remained on the slides after washing. These adherent cells were then immersed in 65 μL of synthetic medium (glucose or galactose as carbon source, depending on the strains), and the culture microchamber was sealed with a coverslip.

Growth on the slides was followed for 8 h, with microscope scanning of an area of 49 mm^2^ every 2 h, (15-min scan per sample), after a first control scan just after spreading and closure of the chamber. For each event of the analyzed population (i.e., the initial single cell or its subsequent microcolony), area (μm^2^) and X, Y coordinates were recorded and compiled in excel files, for further treatment and analysis.

### Data Treatment

Detailed methodology for microcolony growth analysis performed in this project can be found in the Supplementary Methods section in Supplementary Material. In brief, we developed a Python script to calculate individual microcolony growth rates from the experimental datasets (see “individual_cluster_growth_rates_code.zip” in the Supplementary files in Supplementary Material that includes the input data and the Python script called “analysis.py”). The raw experimental datasets include the ID number, area, center X coordinate, and center Y coordinate for each cell cluster (i.e., single cell or microcolony) at each timepoint. As the cluster ID numbers are different at each timepoint, they could not be used to match clusters across timepoints. The first goal of our Python script was therefore to match clusters across timepoints. In most cases, the matching process was straightforward (see Supplementary Figure 2A), and each cluster coordinate set could be simply matched to the nearest coordinate set at the previous timepoint for colony growth rate calculation. Several issues, shown in Supplementary Figures 2B,C, nevertheless required further improvement of the script (see details in Supplementary Methods in Supplementary Material).

The R code used for Hierarchical Cluster Analysis (HCA) of microcolony growth profiles can be found in the Supplementary files in Supplementary Material [see “hierarchical_cluster_analysis.zip” that includes the input data, the R code and an example of data reading and HCA on Ntd-Tsl1 growth rates (Replicate #1)].

For batch cultures in bioreactors, raw OD curves were first treated with time translation (x-axis) to homogenize the initial lag-time and make growth curves as concomitant as possible during their early exponential phase. We then fitted the curves using the *smooth.spline* function of R, which fits a cubic smoothing spline to the supplied data. The smoothing parameter (typically between 0 and 1) was set to 0.4 to keep as natural as possible the main variations of these complex growth curves, yet decreasing noise. Finally, we applied the *predict.smooth.spline* function to calculate the fitted OD at 30 time points equally distributed between 0 and 60 h. The *matplot* function was then used to plot these fitted curves.

## Results

### Study of Yeast Single-Cell Variability of Growth on ConA-Coated Glass-Slides

**Figure 1:**
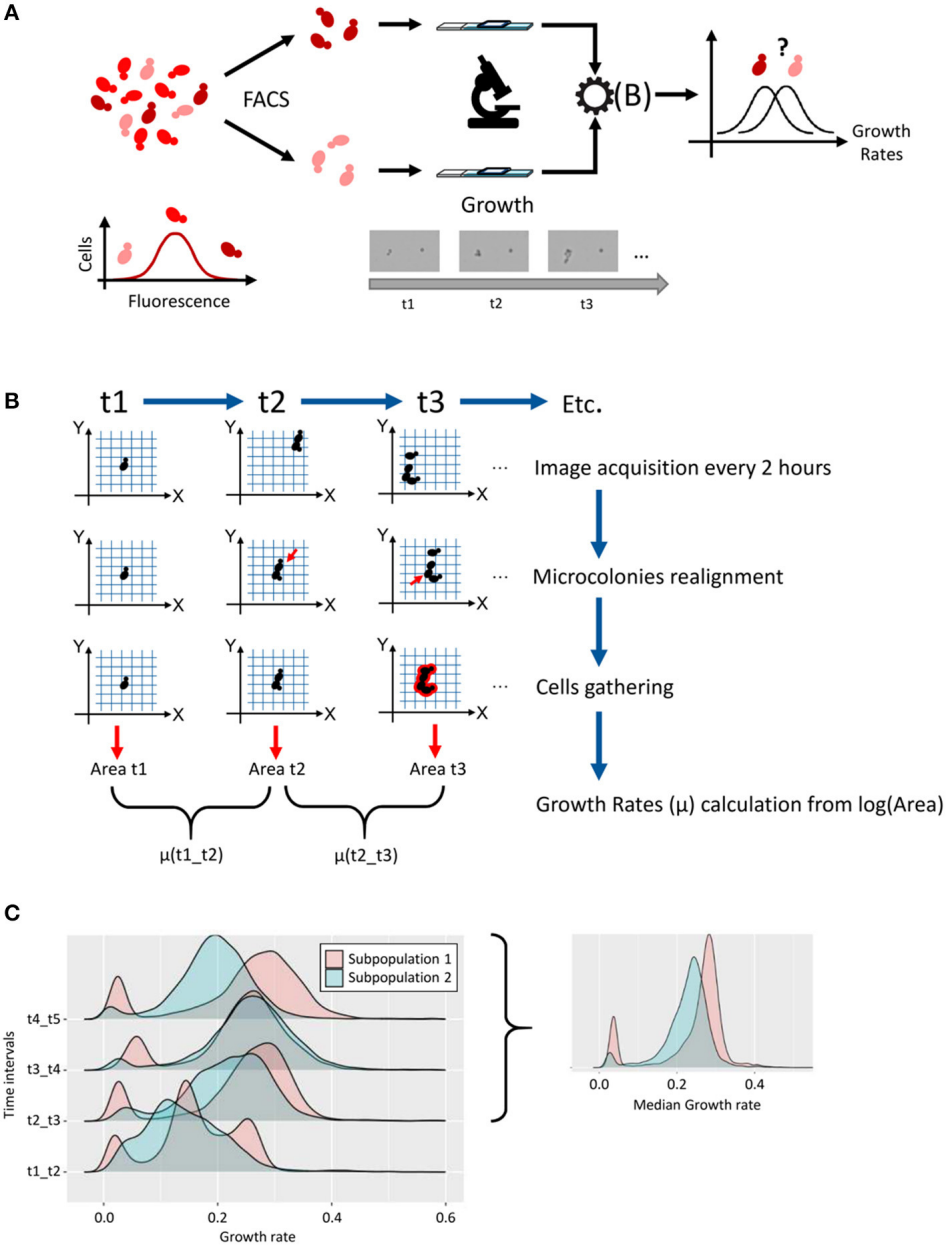
Methodological pipeline for single-cell growth analysis. We developed a methodological pipeline to compare the growth of two subpopulations of cells that are characterized by different levels of expression of a gene of interest. This microscopic device was also used to analyze directly, i.e., without former cell sorting, the growth of strains of interest at the single cell level. **(A)** The first step was to sort cells based on high and low expression of a gene of interest (protein labeled with a fluorescent tag). After sorting, ~5,000 cells were fixed and grown in microchambered slides filled with liquid medium. A scan of the slides was performed every 2 h for 8 h, to follow the growth of every single microcolony. The collected data give us access to several parameters as areas (μm^2^) and XY coordinates on the slides. After data treatment [see **(B)**], the distribution of the growth rate of thousand microcolonies could be plotted and samples compared. **(B)** Data treatment to estimate growth rates. This relied on customized Python scripts that allowed: Colonies realignment, which provided a corrective to the shift of the XY coordinates, due to the manual repositioning of the glass slides under the microscope objective at each time point; Cells gathering to correct splitting of the microcolony; Growth rate calculation between time points, from log(area) values, for each microcolony. **(C)** For each time interval, the distribution of growth rates of thousands microcolonies was presented as density plots. By excluding the first 2 h (initial lag phase, t1–t2 interval) and taking the median value of the three growth rates calculated for the following time intervals, for each microcolony, we obtained a smoothed distribution of growth rate in the sample.

We developed a new methodological pipeline (Figure 1) to follow the growth of thousands of individual yeast cells. For this purpose, we used optical microscopy to scan a large number of cells fixed onto ConA-coated glass slides (Figure 1A). These slides were previously cleaned by a plasma treatment, to increase the hydrophilicity of the surface, hence improving ConA surface functionalization and cell suspension spreading. Immediate image acquisition was essential to determine the initial position of the ~5,000 cells detected on scanned area of the slide. Further scans were then performed every 2 h, for 8 h, keeping the micro-chambers at 30°C between time points for optimal growth conditions.

The raw microscopy datasets included the ID number, area, center X and Y coordinates for each object (i.e., single cell or microcolony) at each time point. The ID numbers were different after each scan, so they could not be used to follow a specific object across time points. Also, the manual repositioning of the glass slides on the microscope at each time point led to a slight shift of the XY positions, with an average of few micrometers, which prevented direct use of this parameter for objects filiation across time points. Our strategy was therefore to merge the datasets from the different time points and use XY position plots to identify cell (or microcolonies) clusters as a function of time (Supplementary Figure 2A). This colony realignment step hence relied on the development of customized Python scripts (see Supplementary Methods in Supplementary Material, analysis.py script).

Adjustments were also required for more complex situations found in some of the datasets. As is shown in Supplementary Figure 2B, the coordinates cannot easily be matched at first glance, and attempting to match them based on the simple method of minimal distance failed. For realignment, we evaluated a cost function for each time point based on how closely it overlays the coordinates of the first time point. We shifted each time point around in each direction and selected the linear adjustment that minimizes the cost function (see Supplementary Methods in Supplementary Material, analysis.py script).

The second adjustment originated from another issue that can interfere with the matching strategy described above. Here, even if time points appeared to be grouped together in well-defined clusters, the problem came from some clusters that had more than one coordinate for a time point, mainly for time point 5 (Supplementary Figure 2C). We could attribute these extra-events to either rare, yet possible dissemination of new-born cells from growing colonies, or to artificial splitting of the signal during image acquisition by the microscope. In both cases, these events were very often separated by a small gap, i.e., less than a micrometer. As these co-localized events might be considered as a single object arising from a single cell after spreading, the script was improved to gather corresponding signals into a single event, which leads to more accurate area estimation relative to the development of the single cell of interest (see Supplementary Methods in Supplementary Material, analysis.py script).

We then used area (μm^2^) measurements as a proxy of cell division and microcolony growth as mentioned in Levy *et al* and described in methods. Growth rates (μ) observed with this method ranged from 0.10 to 0.32 h^−1^ and are consistent with yeast growth rates generally observed in standard flask cultures. In several cases however, a bimodality in growth rate distribution was observed (Figure 1C), with a principal peak corresponding to “growing cells” and a secondary minor peak, which resulted from cells having a much weaker growth than the median growth rate of the population (see below). This observation of slow-growing cells, led us to verify the impact of cell proximity on microcolony development, as previously done in Levy et al. (2012) (see Supplementary Methods and “cluster_distance_code.zip” in Supplementary Material). We therefore counted, for each single cell, the number of neighbors that could be found within a radius of 100 μm. As is shown in the histogram presented in Supplementary Figure 3A for one representative dataset, we noticed that using a cell density of 1,000 cells/mm^2^ during slides preparation, 20% of cells presented 0 or 1 neighbor, while 17% presented five or more neighbors within this area. Interestingly, the good superposition of the cumulative plots of growth rate values for these two categories of cells (Supplementary Figures 3B–D; three different strains taken as representative examples), demonstrated that the proximity of other microcolonies had no significant impact during the first divisions, which was confirmed by the non-parametric Wilcoxon tests used to compare these groups. As a conclusion, we could not associate non- or slow-growing cells to cells with a high number of neighbors after plating.

The heterogeneous distribution of growth rates in each sample of about 5,000 cells led us to perform clustering analysis to highlight different growth patterns. The Hierarchical Cluster Analysis (HCA) method with Euclidean distance was used as a clustering technique on area data as a function of time (see Supplementary Methods in Supplementary Material). The plot of the class center profiles for a typical sample from this study illustrated the diversity of growth patterns within the population (Supplementary Figure 4). We almost systematically observed clusters of slow-growing cells (Supplementary Figure 4, Cluster 5), which represented up to 25% of cells within some samples, yet generally around a few percent in most experiments. These cells, which were associated with a very low growth rate, were located under the left peak in bimodal distributions of growth rates. Other clusters gathered together cells growing with a constant, significant rate, i.e., as a *quasi*-straight line on this log plot (Supplementary Figure 4, Cluster 4). However, many cells also presented an initial lag phase (Supplementary Figure 4, Cluster 17), which could be expected from cells that were transferred in a very different growth environment, here a microculture chamber on glass side, sometimes formerly sorted. Clusters with an initial lag phase during the first 2 h of growth gathered from 37 to 78% of the cells of the samples, which often affected the shape and median growth rate of the distributions on the first time interval, just after spreading (t1 to t2). Hence, to get more representative distributions and reliable estimators of individual growth rates in a sample of interest, the growth rate has been estimated for each microcolony by excluding the first 2 h interval, and taking as final value the median growth rate among the three remaining, almost stabilized values (example in Figure 1C).

### Sorting of Subpopulations on Both Low and High Gene Expression

Isolation of subpopulations with extreme Tsl1 levels, that is low vs. high abundance, was carried out by cell sorting. As *TSL1*-GFP gene fusion was not bright enough to discriminate the fluorescence signal of the low-expressing cells from the autofluorescence background of yeast (Levy et al., 2012) (Supplementary Figure 5A), we used tdTomato which is an improved variant of the red fluorescent protein isolated from *Discosoma* sp. (Shaner et al., 2004) that is brighter than GFP. We found that N-terminal tagging (Ntd-Tsl1 strain) provided a higher fluorescence with about 1-log shift as compared to the GFP tagged strain (Supplementary Figure 5B), which allowed us sorting cells on both high- and low-*TSL1* expression, two subpopulations called, respectively “Plus” and “Minus,” whereas tagging at C-ter of the Tsl1 protein did not. A similar, satisfying result relative to cell-sorting purpose was obtained with the N-terminal fusion of Tps1 with this tdTomato tag (Ntd-Tps1 strain) (Supplementary Figure 5C).

**Figure 2:**
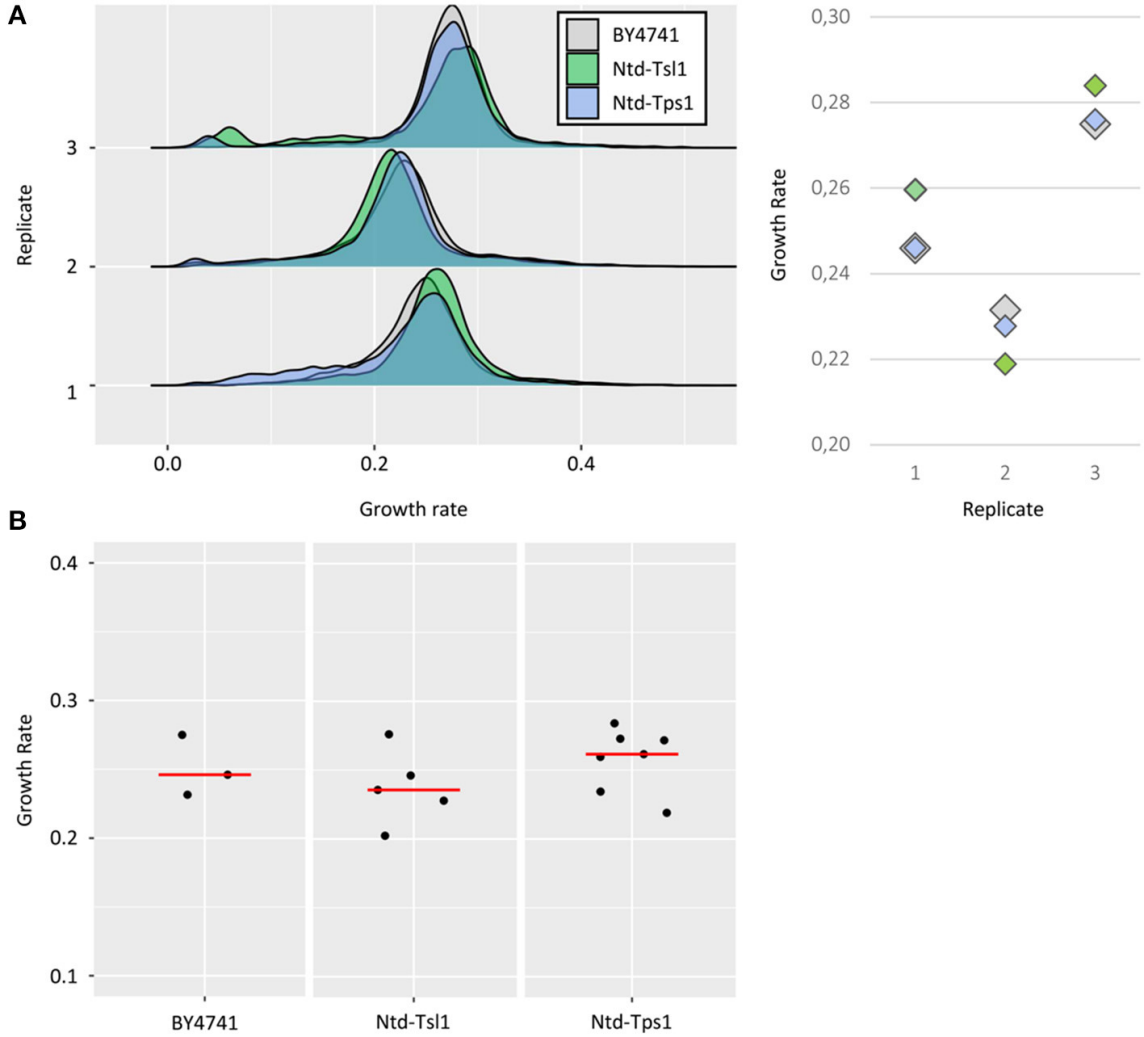
Growth rates of BY4741 and tdTomato tagged strains. **(A)** Growth rate distributions of untagged BY4741 cells (gray), Ntd-Tsl1 cells (green) and Ntd-Tps1 cells (blue) from three replicates. As described in Figure 1C, growth rates were calculated for each single event after excluding the first time interval (lag phase). Median growth rates of the populations ranged from 0,22 to 0,28 h^−1^, with an average SD from the different replicates of ~0,005 h^−1^ (replicate #1 ~0,006 h^−1^, replicate #2 ~0,005 h^−1^ and replicate #3 ~0,004 h^−1^). **(B)** Results from the three independent replicates for the BY4741 strain, the 5 independent replicates for the Ntd-Tsl1 strain and the seven independent replicates for the Ntd-Tps1 strain used to study whether increasing the number of replicates may help reducing the standard deviation and estimating better the growth rate of a strain of interest.

As the main objective of this work was to study growth at the single-cell level, a prerequisite was to show that the fusion of such tags to proteins did not affect the growth rate. The growth of Ntd-Tsl1 and Ntd-Tps1 strains was then compared to the one of WT untagged BY4741 cells. For each of the 3 replicates, no significant differences in median values of the distribution were observed between BY4741 and the tagged strains (average standard deviation (SD) between the three strains of ~0.005, Figure 2A), indicating that these N-terminal fusions could be used as reliable tools for studying growth as a function of *TSL1* or *TPS1* expression. However, this control experiment revealed a low reproducibility between independent experiments carried out at different days, with high variability of median growth rates for a strain of interest, as illustrated by the mean SD of ~0,02, based on a triplicate.

We therefore analyzed whether increasing the number of replicates may help reducing the standard deviation and estimating better the growth rate of a strain of interest, here referred as to the median value of the distribution. By taking independent replicates from this study, that is experiments carried out over different weeks, we could gather three replicates for the BY4741 strain, five replicates for the Ntd-Tsl1 strain and up to seven replicates for the Ntd-Tps1 strain (Figure 2B). The average median growth rates and calculated SD were, respectively 0.251 ± 0,022 h^−1^, 0.238 ± 0,027 h^−1^ and 0.258 ± 0,023 h^−1^. Increasing the number of replicates did not convincingly reduce the SD that was highly dependent on the occurrence of outlier values, as illustrated by the highest SD of the study (0,027) that was obtained from five replicates of the Ntd-Tsl1 strain. The BY strain (three replicates) and Ntd-Tps1 (seven replicates) returned approximately the same SD (0,022 vs. 0,023). By using the mean and SD determined from these three datasets, the confidence intervals for the growth rates at 95% confidence level are respectively (0.196, 0.306), (0.204, 0.271), and (0.236, 0.279). Even in the best situation, random realization of seven replicates for a strain of interest hence gave an estimation of the mean growth rate that is not really satisfying from the yeast physiologist view point (μ±0.02). Unfortunately, lowering this margin to a more acceptable window (e.g., μ ± 0.01) may require more than 20 replicates at 95% confidence level. The origin of this inter-experiment variability is still unexplained, but overall from these data, the much lower SD between experiments carried out in parallel (~0,005 when comparing the three strains) makes the direct comparison between objects of interest possible.

As cell size could influence significantly the cellular amount of tagged proteins under constant gene expression, we applied stringent parameters in terms of cell size and morphology to sort as homogeneous cells as possible (Supplementary Figure 1A). When comparing median sizes right after sorting and spreading of the cells (Supplementary Figure 6A), the ‘Plus’ and ‘Minus’ subpopulations showed median areas of 20.6 ± 1.0 and 17.1 ± 0.8 μm^2^, respectively (mean ± SD over the four replicates). Estimated cell diameters were therefore 5.1 and 4.7 μm, leading to cell volumes of 70 and 53 μm^3^, respectively. This volume ratio of ~1.32 was rather low and could not explain the much higher fluorescence observed in “Plus” cells as compared to ‘Minus’ cells (ratio of ~7) in the sample shown in Supplementary Figure 1B, indicating that the higher abundance of Tsl1 in the “Plus” subpopulation primarily relied on higher expression of the gene.

Finally, the last point that we assessed was the dynamics of the recovery of fluorescence heterogeneity in cell samples, from both the “Plus” and “Minus” subpopulations. Since the measurement of the fluorescence during growth on glass slides was not possible with our microscopy device, we analyzed the stability of the *TSL1* expression in sorted subpopulations by cytometry. After hardly 6 h, the initial distribution was recovered from both subpopulations (Supplementary Figure 6B), whose fluorescence almost perfectly overlapped. However, based on previous data (Levy et al., 2012), we assumed that the initial difference in *TSL1* expression between “Plus” and “Minus” subpopulations could potentially have a short-term, knock-on effect on the microcolony development (see below). From methodological viewpoint, this observation of rapid expression noise recovery led us sorting not less than the extreme 5% of cells from the distribution to reduce the time passed in the collect tube before spreading. Widening the sorting gates at both ends of the distribution was indeed the best compromise, as we wanted to continue using the ‘purity mode’, which is more precise yet slower, settings that altogether allowed sorting 50,000 cells of each type in ~10 min.

### Relationship Between *TSL1* Expression and Growth

**Figure 3:**
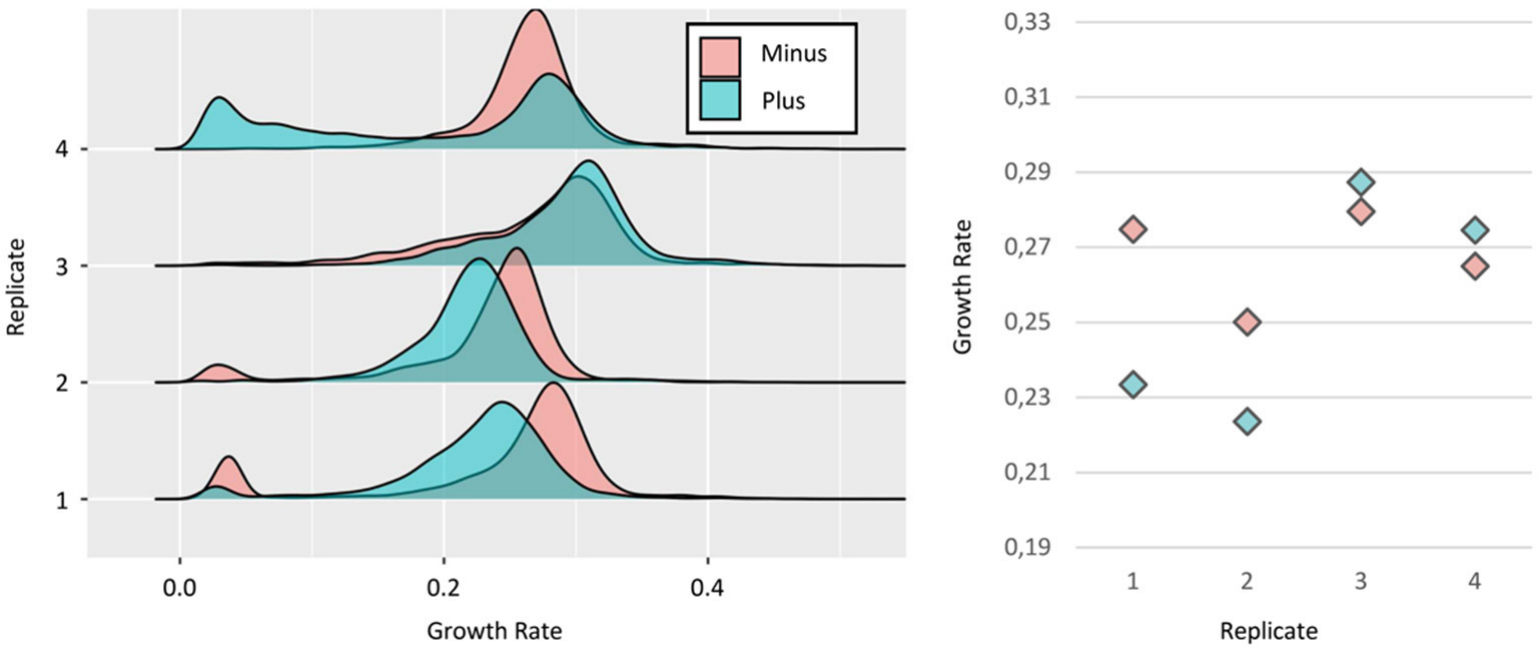
Relationship between *TSL1* expression and growth. (Left) Growth rates distributions of Minus (red) and Plus (blue) sorted subpopulations, from four independent replicates of the strain Ntd-Tsl1. As explained in Figure 1C, the growth rate of each individual microcolony was calculated from three time intervals (t2 to t5). (Right) Median growth rates of the distribution for Minus and Plus cells, respectively: replicate #1 ~0,27 and ~0,23 h^−1^; replicate #2 ~0,25 and ~0,22 h^−1^; replicate #3 ~0,27 and ~0,28 h^−1^; replicate #4 ~0,26 and ~0,27 h^−1^.

We tested this experimental setup firstly on Tsl1 as the expression level of the gene encoding this protein negatively correlated with cell division rate (Levy et al., 2012). As shown in Figure 3, we found a high variability in growth rates between replicates. Especially, we observed that in the first two replicates, the “Minus” cells had higher median growth rates, consistent with published data (Levy et al., 2012). However, the reverse was also observed in replicate #4, while no significant difference could be seen in the replicate #3. Relative to the inter-experiment variability discussed above, we confirmed the high SD on median growth rates for both subpopulations, i.e., 0, 013, and 0.031 for the “Minus” and “Plus,” respectively. About direct, two by two comparison of “Minus” and “Plus” subpopulations in each replicate, the difference could be also significant. As these cells came from the same culture, the source of this variability may arise from intrinsic characteristics of the cells (e.g., expression level). However, the shift that was observed when comparing the “Minus” and “Plus” subpopulations, the one faster than the other and the reverse amongst our four replicates, did not support the existence of a robust physiological determinant of the growth rate of the microcolony, as could have been the initial expression level of genes of interest, that is *TSL1* in this specific case study. Instead, more stochastic, undefined factors seem to fix growth rate distributions. Another notable point that supported this claim was the varying percentage of cells with a slow-growing phenotype, both for the “Minus” and the “Plus” subpopulations, from almost zero up to 14 and 25%, respectively. Interestingly, still in favor of some unpredictable behavior, this phenotype did not systematically co-occur in the subpopulations.

To test whether a more significant change in *TSL1* expression level can influence subsequent growth rate of microcolonies, we replaced the native *TSL1* promoter by the strong promoter of the yeast gene *TDH3*. This choice was driven by a systematic analysis of yeast promoter strength (Peng et al., 2015), where *TDH3* was characterized as one of the strongest promoters in yeast. Cytometry analysis showed that the pTDH3-Tsl1 strain was about 10-times more fluorescent than the strain with the native *TSL1* promoter (Supplementary Figure 7A). Growth of these *TSL1* overexpressing cells was then compared to the one of the wild-type cells after direct spreading of exponentially growing cells on glass slides device. This experiment was duplicated and no clear tendency was observed (Supplementary Figure 7B). On the first replicate, pTDH3-Tsl1 cells were growing faster than control cells with a higher median growth rate of the distribution (0.26 vs. 0.24 h^−1^, Wilcoxon test *p-value* = 9.1 e^−76^). On the second replicate, pTDH3-Tsl1 cells contrarily grew much slower than control cells (0.17 h^−1^ vs. 0.24 h^−1^, Wilcoxon test *p-*value = 1.8 e^−75^). To conclude, although often significant from a statistical viewpoint due to the huge number of microcolonies that were analyzed, the differences that fluctuate sometimes in one direction and sometimes in the other, when comparing either two extreme subpopulations or overexpressing vs. WT strains, suggest that the initial *TSL1* expression level in a cell does not influence the subsequent growth rate of the microcolony.

### Relationship Between *TPS1* Expression and Growth

**Figure 4:**
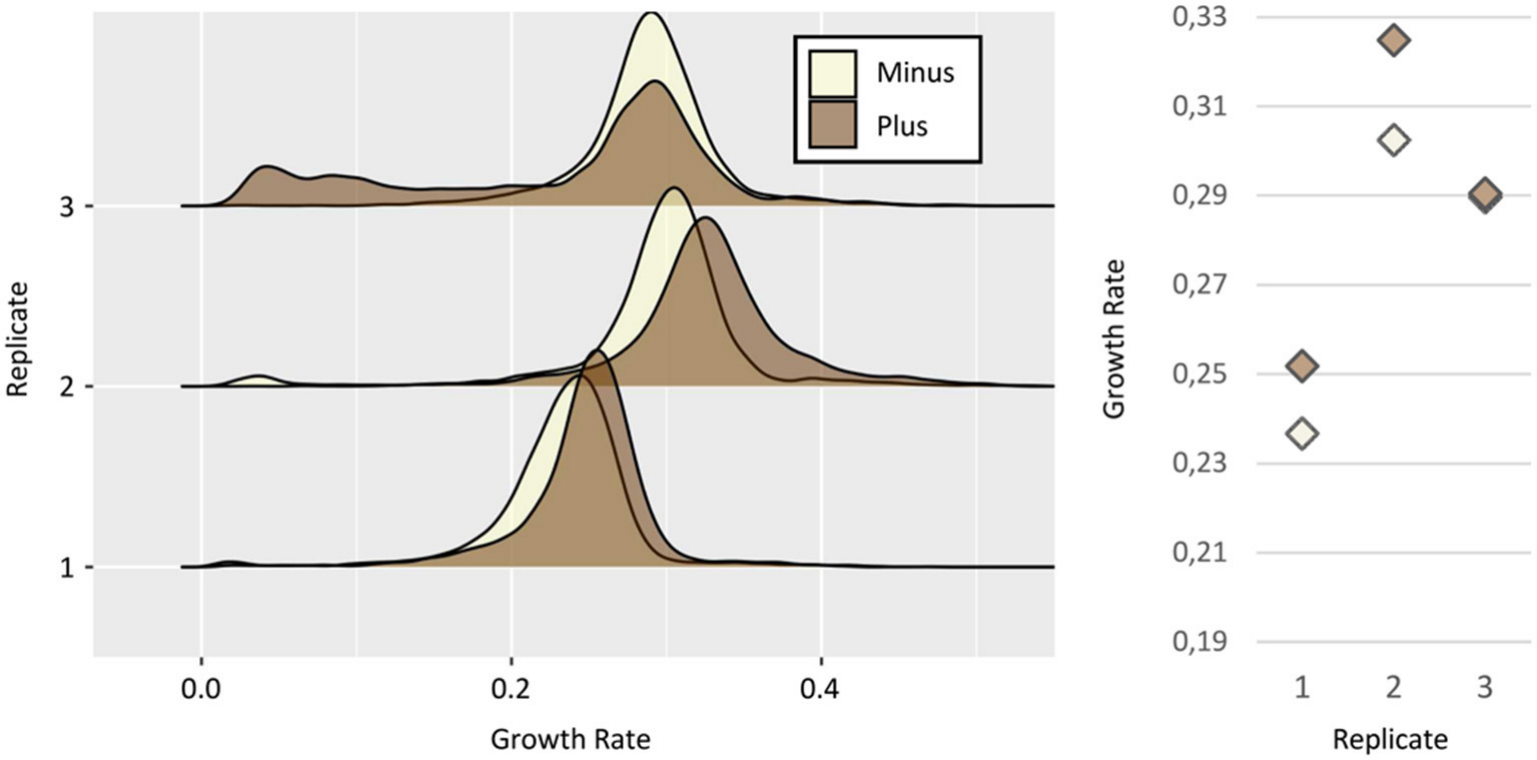
Relationship between *TPS1* expression and growth. (Left) Growth rates distributions of Minus (white) and Plus (brown) sorted subpopulations, from three independent replicates of the strain Ntd-Tps1. Growth rates calculation as in Figure 3. (Right) Median growth rates of the distribution for Minus and Plus cells, respectively: replicate #1 ~0,23 and ~0,25 h^−1^; replicate #2 ~0,30 and ~0,32 h^−1^; replicate #3 ~0,28 and 0,29 h^−1^.

As Tps1 was also mentioned as a potential marker of growth heterogeneity in yeast populations (Levy et al., 2012), we used the same approach with a strain harboring a tdTomato-*TPS1* gene fusion under the control of the native *TPS1* promoter, to sort the “Plus” and “Minus” subpopulations (Figure 4). We first showed that the fluorescence of the Ntd-Tps1 strain did not overlap with the autofluorescence background of the wild-type strain (Supplementary Figure 5C), allowing efficient cell sorting of both “Minus” and “Plus” subpopulations. Moreover, the growth rate of this tagged strain was the same as the WT BY4741 strain (Figure 2A), after direct spreading of exponentially growing cells on the slides and their incubation in a glucose medium. Taking into account that mutation in the *TPS1* gene, including its deletion, may cause growth alteration (Thevelein and Hohmann, 1995; Gancedo and Flores, 2004), this result indicated that the strain bearing the tagged Tps1 version behaves like the WT strain.

Growth of the sorted subpopulations was then analyzed (Figure 4). We observed a statistically significant advantage of growth for the “Plus” cells in two of the three replicates, i.e., 0.25 vs. 0.23 h^−1^ (Wilcoxon test *p-*value = 6.1 e^−117^) and 0.32 vs. 0.30 h^−1^ (Wilcoxon test *p-*value = 6.9 e^−178^), for replicates #1 and #2, respectively. The third one showed a more complex situation as the statistically significant advantage of growth for the “Plus” subpopulation (Wilcoxon test *p-value* = 0.0245) was quite insignificant in biological terms (0.290 h^−1^ vs. 0.289 h^−1^). However, as it was already reported for Tsl1 experiments, the strong inter-replicates variability of the median of growth rate distributions, ranging from 0.23 to 0.32 h^1^ (Figure 4), suggested that these small differences between subpopulations probably reflect technical bias, more than a true biological effect.

**Figure 5:**
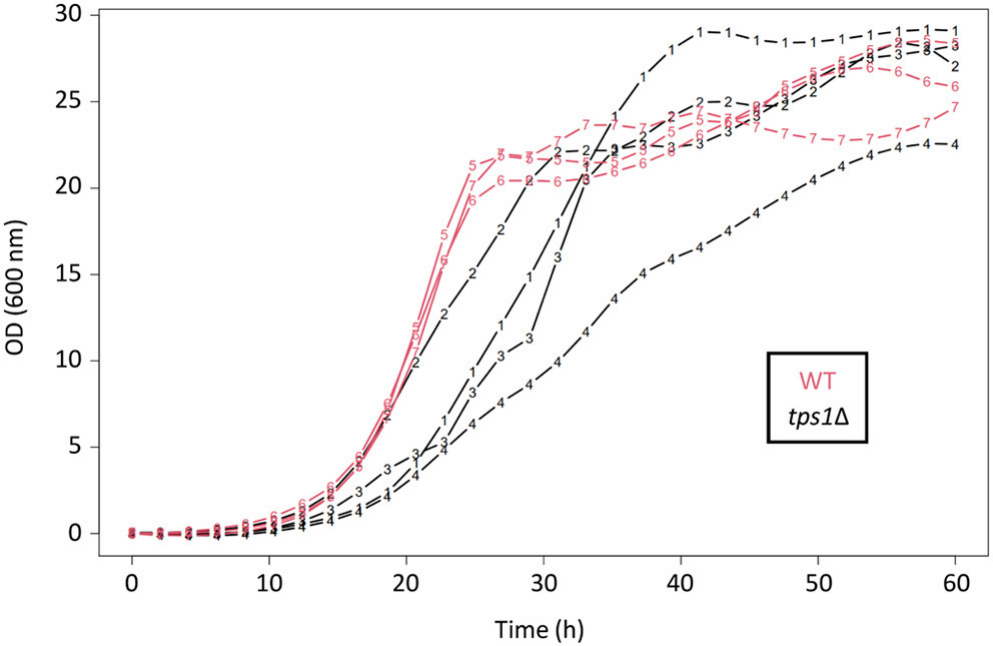
Batch cultures of the *tps1*Δ mutant in controlled bioreactors. OD (600 nm) values as a function of time (hours), for four independent replicates of the *tps1*Δ strain (black; 1–4) and three replicates of the WT strain (red; 5–7). These strains were grown for several days on galactose minimal medium, in controlled bioreactors. Both are prototrophic CEN.PK strains. The WT replicates showed a typical diauxic growth, with a metabolic shift around 25 h. For the sake of clarity, raw OD curves were fitted as described in methods.

The *TPS1* gene is nevertheless singular, its deletion harboring characteristic phenotypes including an inability to grow on fermentable sugars (Thevelein and Hohmann, 1995; Gancedo and Flores, 2004; Walther et al., 2013). This is the reason why this mutant is currently cultivated on galactose as permissive carbon source. Interestingly, cultures of this *tps1*Δ strain showed non-predictable growth patterns even when performed in controlled bioreactors (unpublished growth experiments from our lab). Indeed, batch cultures on galactose of the *tps1*Δ cells led to high variability between replicates, as compared to the WT strain that exhibited more reproducible diauxic growth in terms of shape and quantitative parameters (Figure 5).

**Figure 6:**
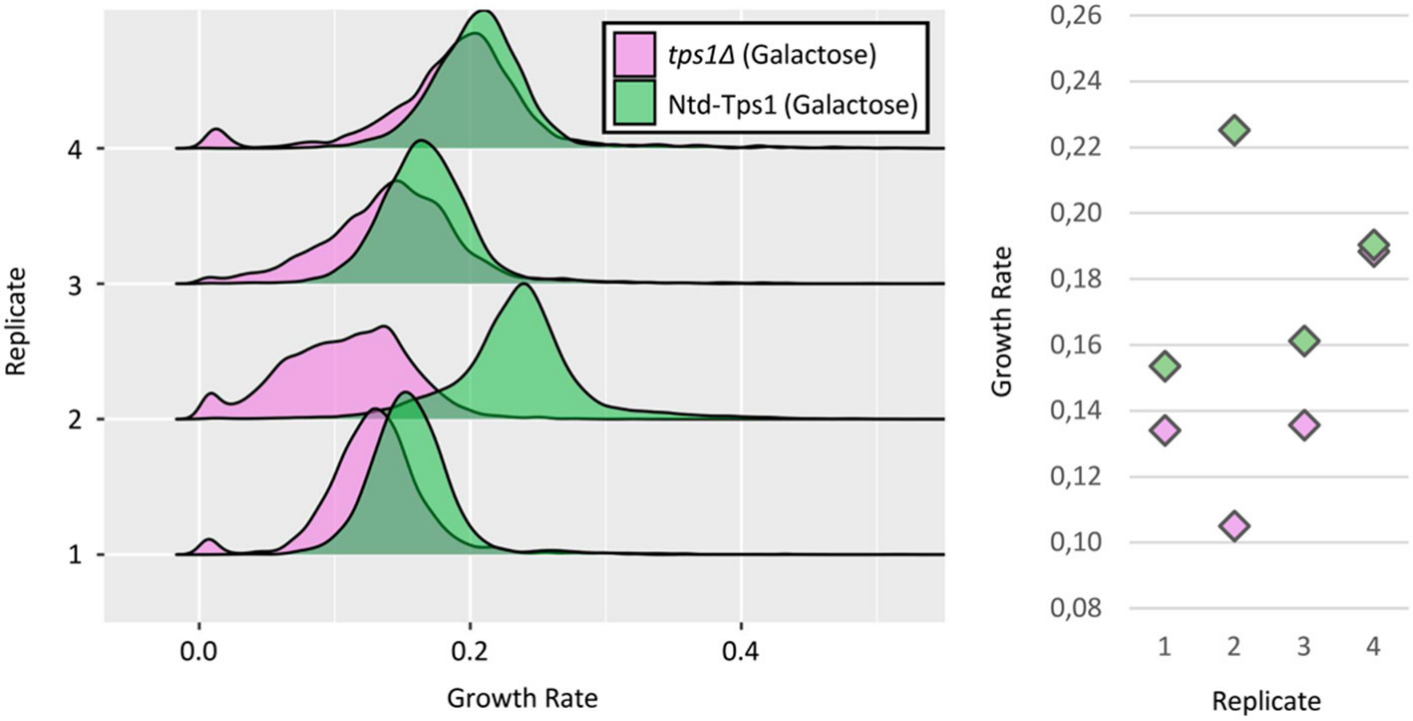
Growth phenotype of the *tps1*Δ strain at the single-cell level. (Left) Growth rates distributions of *tps1*Δ cells (pink) and control, Ntd-Tps1 cells (green), with galactose as carbon source, from four independent replicates. Growth rates calculation as in Figure 3. (Right) Median growth rates of the distribution for *tps1*Δ and Ntd-Tps1 cells, respectively: replicate #1 ~0,13 and ~0,15 h^−1^; replicate #2 ~0,10 and ~0,22 h^−1^; replicate #3 ~0,13 and ~0,16 h^−1^; replicate #4 ~0,18 and ~0,19 h^−1^.

Then we asked whether the results obtained at the single-cell level are relevant and help understanding behavior at the population level. For that purpose, we investigated its growth phenotype at the single-cell level. Over the four replicates, the *tps1*Δ strain harbored a reproducible, slower growth phenotype as compared to the WT strain (Figure 6). Interestingly, *tps1*Δ cells showed an important and variable proportion of slow-growing cells (5.97% ± 2.89), which was never observed for the WT strain on galactose (Figure 6). The most noticeable point was nevertheless the variable shape of the main peak that contains growing cells. It can appear as a distribution similar to the one of the WT strain (e.g., replicate #1), but also under wider and much more irregular distributions, which characterized a huge heterogeneity in the growth rates of individual cells. Thus, the great variability of growth patterns and the slower growth tendency of the *tps1*Δ strain at the population level, may find explanation at the single-cell level. Both the variable proportion of slow-growing cells between replicates, and the highly heterogeneous population of growing cells in terms of individual growth rates, may indeed contribute to the unstable growth behavior of this mutant strain in batch cultures.

## Discussion

Technological advances in recent years led to insights in the differences between fast and slow-proliferating cells in microbial populations and tried to identify markers for proliferation rate at the single-cell level (Levy et al., 2012; Ziv et al., 2013, 2017; Van Dijk et al., 2015; Cerulus et al., 2016; Li et al., 2018; Dhar et al., 2019). To help deciphering how yeast single-cell variability of growth is controlled, we developed in this work a new methodological pipeline that allowed single-cell growth analysis after FACS. We applied stringent criteria on morphological aspects during cell sorting, which allowed us limiting variability due to differences in cell states and studying homogeneous cell populations. From statistical viewpoint, thanks to the huge size of samples (i.e., several thousand events), significant results were generally observed in most of the growth experiments, either between strains or between subpopulations from one strain. However, the observed differences of growth should be considered with caution, especially when considering the high variability between biological replicates for a given strain, which could be indicative of a day-specific stress on the pre-culture before sorting, or during incubation of the slides that nevertheless evolved in parallel. More importantly, reversals of the situation for these comparisons when replicating the experiment, i.e., the one faster than the other and the reverse amongst the replicates, minimized the relevance of these statistically significant differences in the two by two comparisons.

A limitation of our tool was also the impossibility to check the expression status of the cells during the growth of the microcolonies, because of the lack of fluorescence detection in our microscopy system. To circumvent this last limitation, we nevertheless performed a recovery experiment showing that the initial difference in Tsl1 expression was rapidly recovered after <6 h. This result was different from the one obtained for other genes such as *SIR2* (Liu et al., 2020) for which the difference in expression was mostly maintained after 6 h, the initial heterogeneous fluorescence distribution being recovered after 24 h. Those measurements indicated the *TSL1* expression level is quite unstable overtime. This observation was already mentioned in Levy et al., while not quantified (Levy et al., 2012). Indeed, these authors reported that from initially low-expressing cells, the switching from low to high expression can occur very rapidly in the first 4 h or later in some cells of microcolonies that had already grown (Levy et al., 2012). They therefore concluded that the growth was mainly associated to low *TSL1* expression. However, analyzing the correlation between the average expression level in the final microcolony and the size of the microcolony as performed by Levy et al. is not adequate to determine if the initial *TSL1* expression level determines the future growth rate. The strategy that we adopted in this study was different because cells were first discriminated depending on their expression level and it was probably more adapted to measure the real impact of the initial expression on growth. The rapid switching of *TSL1* expression does not allow to conclude on such a short or mid-term impact on growth rate, particularly to find the negative correlation that was previously published.

The discrepancy between our results and those from Levy et al. (2012) could be also linked to cell sorting by cytometry. Indeed, sorting cells among a very homogeneous population in terms of morphological criteria probably limits confounding effects that take place at the whole population level. By excluding most of the effects linked to differences in cell size and other physiological differences, we mostly restrict the sources of cell-to-cell heterogeneity to the so-called intrinsic noise (due to the random molecular events that occur for each gene independently). Concentrating on variations solely due to these random molecular events might exclude phenomena that produce a great part of the *TSL1* expression variability and contribute to most of its correlation with growth rate. This correlation may not exist when considering primarily intrinsic noise. Finally, evidence for a lack of correlation was reinforced by the *TSL1* overexpression experiment that did not generate any negative effect on growth.

Additionally, without searching for a causal relationship between *TSL1* expression level and growth rate, recent works instead attempted to decipher the common pathways that control both phenomena and may lead to anti-correlation. Li et al. (2018) showed that natural variations in the Tsl1 cellular amount were especially linked to heterogeneity in cAMP levels that may trigger cellular heterogeneity in the activities of the stress-associated transcription factors Msn2 and Msn4. This in turn would be at the origin of both cell-to-cell variability of growth rate and stress tolerance, Tsl1 expression being considered here as a stress marker. While Msn2 was also required for both heterogeneous expression of Tsl1 and slow growth, Msn4 was only required for normal abundance of slower-growing cells and not for heterogeneous Tsl1 expression, suggesting that slow growth and stress tolerance are not inevitably linked. Moreover, there were both Msn2-dependent and -independent slower-growing cells, suggesting that slow-growth can also originate from mechanisms that are not mediated by Msn2 transcription factor (Li et al., 2018). Thus, depending on the weight of these different mechanisms in the generation of slow-growth, the variable Msn2/Msn4-associated expression of *TSL1* from cell-to-cell could not be necessarily associated with single-cell variability of growth if non-stress-related mechanisms were predominant, and partly explain the high variability we observed between replicates.

Cellular amounts of other members of the TPS complex were also suggested to be associated with single-cell variability of growth (Levy et al., 2012). Among them, Tps1 is particularly interesting because it could be a key component of yeast metabolism, with possible alternative functions apart from its essential role in trehalose synthesis (Walther et al., 2013; Van Heerden et al., 2014). However, as for Tsl1, natural variations in the initial abundance of Tps1 did not produce single-cell growth variability, as did cellular processes such as cell signaling through cAMP (Li et al., 2018), energetic metabolism with the TCA cycle (Ziv et al., 2017) and sugar transport (Cerulus et al., 2016).

Nevertheless, we further investigated the behavior at the single-cell level of the mutant defective of *TPS1* due to its singular phenotypes such as lack of growth on glucose (Walther et al., 2013; Van Heerden et al., 2014). Another interesting phenotype of this mutant strain is the highly variable growth pattern in batch culture experiments, even on permissive carbon sources. It is interestingly to stress that the experiments that were reported in this work have been performed with strains from the CEN.PK family that has been selected and increasingly used as offering an acceptable compromise between the criteria set by different yeast research disciplines, including the robustness of its growth kinetics (Van Dijken et al., 2000). At the single-cell level, the highly variable proportion of slow-growing cells that were not present in a WT strain, together with more heterogeneous growth rates of individual growing cells, might partly generate the highly variable behavior of this *tps1*Δ mutant at the whole population level, which had been observed in controlled bioreactors. Thus, we showed once again the interest of single-cell analysis to decipher complex and sometimes stochastic phenotypes at the population level.

## Data Availability Statement

The original contributions presented in the study are included in the article/Supplementary Material, further inquiries can be directed to the corresponding author/s.

## Author Contributions

J-LP and J-PC conceived and designed the experiments. SA acquired the data. MS-A and CM-R treated the growth data. SA, J-LP, and J-PC interpreted the data and wrote the manuscript. AS and J-MF supported the project and reviewed the manuscript. All authors contributed to the article and approved the submitted version.

## Conflict of Interest

The authors declare that the research was conducted in the absence of any commercial or financial relationships that could be construed as a potential conflict of interest.
