# Supplementary material for: Insights on the Control of Yeast Single-Cell Growth Variability by Members of the Trehalose Phosphate Synthase (TPS) Complex
Source: Front Cell Dev Biol. 2021 Jan 28;9:607628. doi: 10.3389/fcell.2021.607628 (PMC7876269; doi:10.3389/fcell.2021.607628)
Supplement: Supplementary file 1 [file Table_1.DOCX]

Supplementary Material

Insights on the control of yeast single-cell growth variability by members of the Trehalose Phosphate Synthase (TPS) complex

Sevan Arabaciyan, Michael Saint-Antoine, Cathy Maugis-Rabusseau, Jean Marie François, Abhyudai Singh, Jean-Luc Parrou and Jean-Pascal Capp^*^

*** Correspondence:** [capp@insa-toulouse.fr](mailto:capp@insa-toulouse.fr)

**Supplementary Figure 1.** Cell sorting parameters. (A) (Left) Forward- and side-scatter density plots, displaying the Ntd-Tsl1 cell population and a first gate applied for excluding debris. (Middle) Forward-scatter plot from height values and area values with a second gate excluding non-single cells. (Right) Side-scatter plot from previous gate, with height values and area values, with a third gate excluding non-single cells on a different parameter. (B) Fluorescence level distribution from Ntd-Tsl1 cells (blue) during exponential growth phase and the sorting gates labelled ‘Minus’ and ‘Plus’ respectively.

**
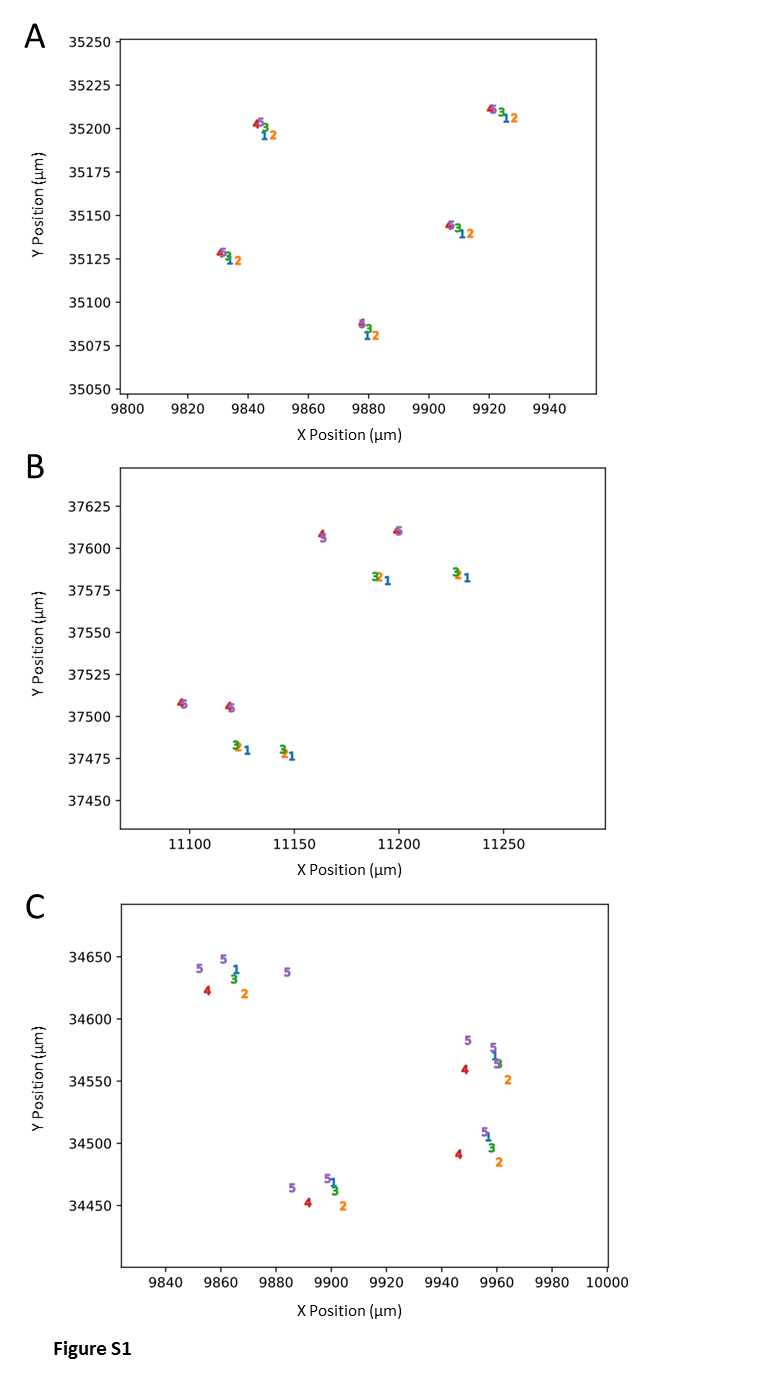
**

**Supplementary Figure 2.** Spatial analysis and growth rate calculation. (A) Here a basic time point clustering is sufficient for correlation of areas evolution. (B) Subsection of one *tps1Δ* cells dataset, with time point 4 and 5 that present a shift as compared to time points 1, 2 and 3 that perfectly clustered. A homemade script was necessary to realignment of shifted time point. (C) Subsection of one Ntd-Tps1 Plus subpopulation cells dataset, with time point 5 that exhibits from 1 to 3 independent objects for a single microcolony. A gathering of events from correlated time point unable correction of disseminating cells overtime.

**
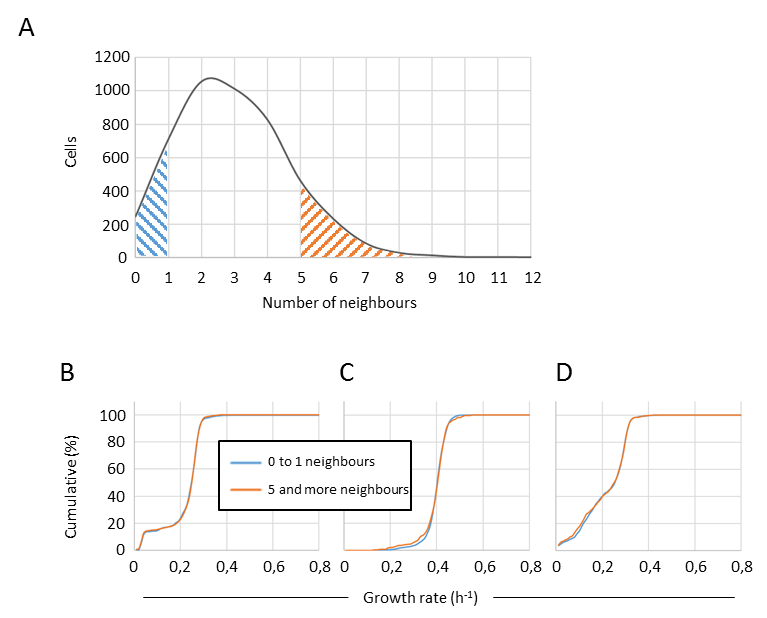
**

**Supplementary Figure 3.** Impact of cells proximity on growth rate. (A) The histogram reports for each cell of the slide, the number of neighbours within a radius of 100 µm (here, a representative sample of approx. 5,000 cells (Strain Ntd-Tsl1, ‘Minus’ subpopulation, replicate 1)). (B-D) Cumulative plots for cells that have 0 or 1 neighbour (blue curve), and cells that have 5 or more neighbours (orange curve); (B) strain Ntd-Tsl1, ‘Minus’ subpopulation, replicate 1, (C) strain Ntd-Tps1, ‘Plus’ subpopulation, replicate 1 and (D) strain *tps1Δ*, replicate 2. The growth rate of each cell/microcolony was calculated here by using the increase in area between T1 (spreading) and T5 (8 hours later). The non-parametric Wilcoxon tests between the two groups gave p-values of 0.43 (B), 0.94 (C), and 0.83 (D), indicating no significant difference.


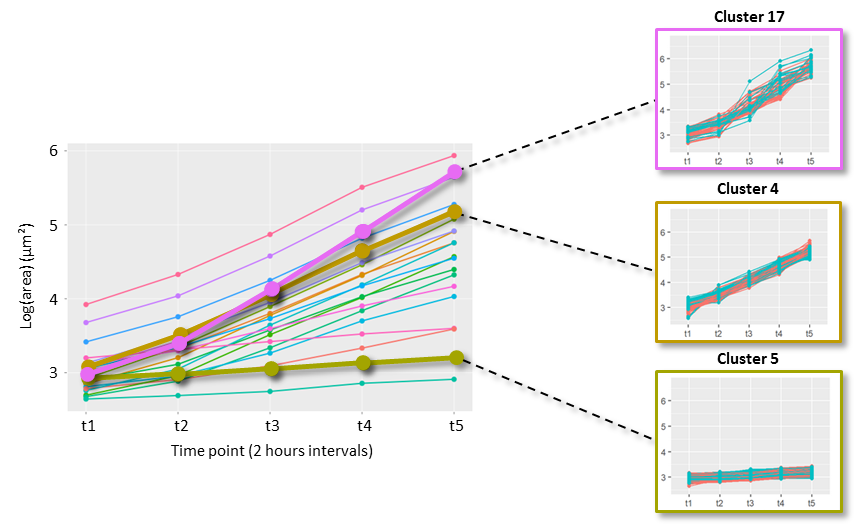


**Supplementary Figure 4.** Clustering to highlight the different growth regimes in a population of cells. Hierarchical cluster analysis (HCA) set for 20 clusters, of a representative sample (strain Ntd-Tsl1, replicate 1, approx. 9000 cells). The left plot shows the evolution of log(areas) as a function of time, for each class center. On the right, the plots show the area evolution profile of each cell belonging to a given cluster. The 3 selected clusters are representative of 3 growth patterns in a yeast population, i.e. slow-growing (Cluster 5), constant growth (Cluster 4) and lag-phase (Cluster 17).


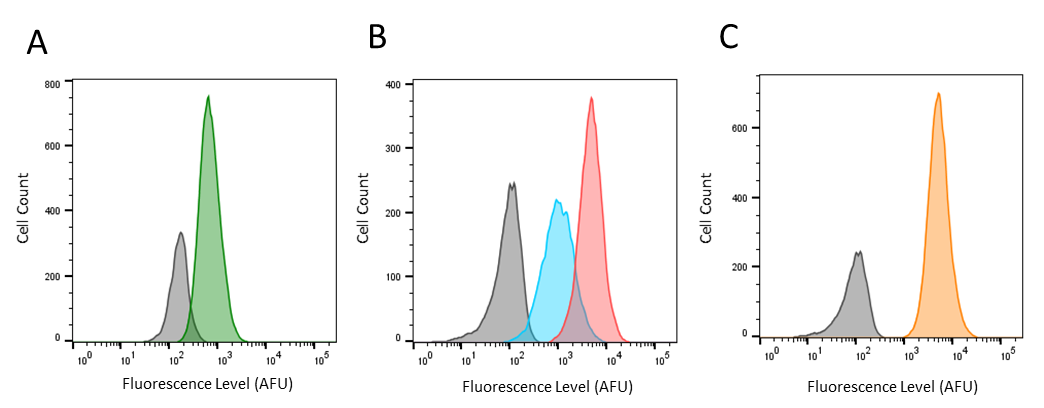


**Supplementary Figure 5.** Fluorescence signal from GFP and tdTomato tags. Histograms of fluorescence level (AFU) from cytometry analysis. (A) *TSL1* gene tagged with GFP (green), BY4741 autofluorescence (grey). (B) tdTomato tag at the N-terminus (Ntd-Tsl1 strain, red) and C-terminus (Ctd-Tsl1 strain, blue) of the Tsl1 protein; BY4741 autofluorescence (grey). (C) tdTomato tag at the N-terminus of the Tps1 protein (Ntd-Tps1 strain, orange) and BY4741 autofluorescence (grey).

**Supplementary Figure 6.** Cells size and *TSL1* expression correlation. (A) Areas distribution for Minus (red) and Plus (blue) sorted subpopulation from Ntd-Tsl1 cells over 4 replicates at the first time point measurement, right after sorting. A ratio of 1,32 was measured between Minus and Plus areas, compared to the approx. 7 log gain in fluorescence level. (B) After sorting the ‘Plus’ (red) and ‘Minus’ (blue) subpopulations, fluorescence measurement was followed every 2 hours. The histogram represents the fluorescence level (AFU) of these 2 subpopulations, 6 hours after sorting procedure.

**Supplementary Figure 7.** Effect of *TSL1* overexpression. (A) Fluorescence level (AFU) from the Ntd-Tsl1 (blue) and pTDH3-Ntd-Tsl1 (orange) strains. BY4741 strain (grey) as control cells for autofluorescence. (B) Growth rates distributions, from 2 replicates of overexpressing pTDH3-Tsl1 cells and the control strain Ntd-Tsl1 (native promoter). Growth rates calculation as in Figure 3.

**Supplementary Table 1.** List of strains used in this study.

**Supplementary Table 2.** List of primers used in this study.

**Supplementary Methods.**

*Calculation of Cluster Growth Rates*

We developed a Python script to calculate individual cluster growth rates from the experimental datasets (see “individual_cluster_growth_rates_code.zip” in the supplementary files that includes the input data and the Python script called “analysis”). The raw experimental datasets include the ID number, area, center X coordinate, and center Y coordinate for each cell cluster at each timepoint. The goal of our Python script is to match clusters across timepoints, so that we can calculate the growth rate of each individual cluster. The cluster ID numbers are different at each timepoint, so they cannot be used to match clusters across timepoints. Instead, proximity must be used.

In most cases, the matching process was straightforward (see Figure S2A), and each cluster coordinate set could be simply matched to the nearest coordinate set at the previous timepoint. However, there were two issues we sometimes encountered, which we will refer to as the alignment issue and the cluster breakup issue. In the alignment issue (see Figure S2B), one or more of the timepoints have a linear displacement from the rest of the timepoints, and must be realigned before the clusters can be matched using the simple minimal distance method. In the cluster breakup issue (see Figure S2C), some cell clusters have become large enough that they are recorded as two timepoint coordinate sets, so these must be combined together before they can be matched using the minimal distance method.

For each dataset we have five CSV files, labeled “t1”, “t2”, “t3”, “t4”, and “t5” for each timepoint. Each CSV contains four columns: cluster ID, area, center X position, and center Y position. We begin by reading in the t1 and t2 CSVs and storing them as two-dimensional NumPy arrays. Next, we use our alignment() function to check for the alignment issue described in the previous paragraph, and correct it if needed. The alignment() function applies linear shifts in each direction to the t2 coordinate sets. For each possible linear shift, a cost function is then evaluated to score the distance between each of the shifted t2 coordinate sets and their nearest t1 coordinate set. If there is a linear shift that yields a lower cost (meaning better alignment) than the original position of the t2 coordinates, then this linear shift is applied to the t2 coordinates to be kept going forward. The linear shifting is done within a pre-defined search space. The default search space in our code is a 40 unit by 40 unit square around the original t2 position, but if this fails to fix an alignment issue then it may be prudent to use a larger search space, such as 100 unit by 100 unit, or 200 unit by 200 unit, although this will increase the runtime of the code significantly.

Although the logic behind our alignment() function is sound, we also inspected the results visually, to be safe, in cases where this function resulted in a correction being applied. In these cases, we plotted a superposition of the coordinates at both timepoints, similar to the coordinate superposition is Figure S2. We then checked to confirm that there was a linear displacement occurring between t1 and t2 that was corrected after the alignment() function was applied.

After ensuring that the t1 and t2 coordinates are properly aligned, we can then begin matching t2 clusters to t1 clusters. To do this, we first define a Python list object called “matches”. Then, for each t2 cluster, we compute the distance between it and every t1 cluster. We then choose the t1 cluster with the minimum distance as the match of each t2 cluster, and record this match in the matches list. This process is done using a nested for-loop, and by the end each t2 cluster is matched with its nearest t1 cluster.

In some unusual cases, more than one t2 cluster could be matched to the same t1 cluster. When we encountered this situation, we visually inspected it by plotting superpositions of the t1 and t2 clusters. In some very rare cases, we found that the spatial layout was simply incoherent and the entire dataset had to be thrown out. However, in other cases duplicates of one timepoint clearly corresponded to a certain cluster (please see Figure S2C for an example). In these cases, we made the assumption that the duplicate coordinates were part of the same cluster, which had been broken up in the measurement, possibly due to large size. In these cases, the areas of the duplicates were added together, and a new coordinate center was assigned by taking the K-means center of the coordinates, using the Python sklearn.cluster Kmeans() function.

Next, we go through the matches list and delete any rows for which the t2 and t1 matching clusters do not fall within a distance threshold of 25 units (for more spaced out datasets this threshold may be increased at the user’s discretion). This step is to ensure the quality of our matches. However, if too many rows end up being deleted, the dataset should be visually inspected to check its quality and coherence.

At this point, we have a one-to-one mapping of t2 clusters to t1 clusters. Next, we compute the growth rate for each cluster by plugging the areas into the formula $\frac{\log\left( \frac{A_{t+1}}{A_{t}} \right)}{2}$ where $A_{t}$ is the area at t1 and $A_{t+1}$ is the area at the t2. We use natural logarithm for this calculation, and divide by 2 since the timepoints are 2 hours apart from each other. Finally, the information for each match, including the computed growth rate, is saved in a new variable called “t1_t2”.

The previous several paragraphs have described the process of matching t2 clusters to t1 clusters and computing their growth rates. Next, we apply this same process to match t3 clusters to t2 clusters, t4 clusters to t3 clusters, and t5 clusters to t4 clusters. The results are saved in list objects called “t2_t3”, “t3_t4”, and “t4_t5”.

Finally, we must string together all of the matches across all five timepoints. This is done using the cluster ID numbers for the overlapping timepoints. For example, clusters in the t1_t2 list are matched to the clusters in the t2_t3 list using the t2 cluster ID numbers. This is done across all of the timepoints using four nested for-loops. We then write two output CSVs, one with the areas of each match at each timepoint, and one with the growth rates between each timepoint for each match.

Our code, called “analysis.py”, is available online, and we hope that it will be useful for researchers working on similar projects. For questions and technical support, please contact Michael Saint-Antoine at [mikest@udel.edu](mailto:mikest@udel.edu).

*Cluster Proximity Analysis*

In a preliminary analysis, we wanted to see if the growth rates of clusters were affected by the clusters’ proximity to each other. To do this, we developed a Python script called “cluster_distance_analysis.py” (see “cluster_distance_code.zip” in the supplementary files). This script matches clusters across timepoints in a similar way to the method described in the previous section, except t1 clusters are matched directly to t5 clusters, and growth rates are computed for this 8-hour period, rather than across 2-hour increments. Also, this preliminary analysis was done before we encountered the alignment and cluster breakup issues, so the corrections for these issues were not used in this code.

After clusters have been matched across timepoints and growth rates have been calculated, we then assigned an ID number to each cluster match. Next, we added a column to each cluster match row, where we list all other cluster matches within a distance of 100 units. Finally, all of this information is written to an output CSV.

*Hierarchical Cluster Analysis (HCA)*

See “hierarchical_cluster_analysis.zip” in the supplementary files that includes the input data, the R code used, and an example of data reading and HCA (on the Ntd-Tsl1 growth rates, Replicate #1) in the file “Script_Clustering_GrowthData.docx”.
