# Supplementary material for: Insights on the Control of Yeast Single-Cell Growth Variability by Members of the Trehalose Phosphate Synthase (TPS) Complex
Source: Front Cell Dev Biol. 2021 Jan 28;9:607628. doi: 10.3389/fcell.2021.607628 (PMC7876269; doi:10.3389/fcell.2021.607628)

Clustering results exemple on Ntd-Tsl1 Growth Rates Replicate 1

September, 2020

Table of Contents

[Datas reading 4](#_Toc51250107)

[Clustering (CAH) 10](#_Toc51250108)

[Ward 10](#_Toc51250109)

[For 20 Clusters 11](#_Toc51250110)

knitr::opts_chunk$set(warning=FALSE,message=FALSE)

library(stringr)
library(tidyverse)

## Warning: package 'tidyverse' was built under R version 3.6.1

## -- Attaching packages ------------------------------------------------------------------------------------------------------------- tidyverse 1.2.1 --

## v ggplot2 3.2.1 v readr 1.3.1
## v tibble 2.1.3 v purrr 0.3.2
## v tidyr 0.8.3 v dplyr 0.8.1
## v ggplot2 3.2.1 v forcats 0.4.0

## Warning: package 'ggplot2' was built under R version 3.6.1

## -- Conflicts ---------------------------------------------------------------------------------------------------------------- tidyverse_conflicts() --
## x dplyr::filter() masks stats::filter()
## x dplyr::lag() masks stats::lag()

library(plotly)

## Warning: package 'plotly' was built under R version 3.6.1

##
## Attaching package: 'plotly'

## The following object is masked from 'package:ggplot2':
##
## last_plot

## The following object is masked from 'package:stats':
##
## filter

## The following object is masked from 'package:graphics':
##
## layout

library(ggplot2)
library(gridExtra)

##
## Attaching package: 'gridExtra'

## The following object is masked from 'package:dplyr':
##
## combine

library(plyr)

## -------------------------------------------------------------------------

## You have loaded plyr after dplyr - this is likely to cause problems.
## If you need functions from both plyr and dplyr, please load plyr first, then dplyr:
## library(plyr); library(dplyr)

## -------------------------------------------------------------------------

##
## Attaching package: 'plyr'

## The following objects are masked from 'package:plotly':
##
## arrange, mutate, rename, summarise

## The following objects are masked from 'package:dplyr':
##
## arrange, count, desc, failwith, id, mutate, rename, summarise,
## summarize

## The following object is masked from 'package:purrr':
##
## compact

library(reshape2)

##
## Attaching package: 'reshape2'

## The following object is masked from 'package:tidyr':
##
## smiths

library(mixOmics)

## Loading required package: MASS

##
## Attaching package: 'MASS'

## The following object is masked from 'package:plotly':
##
## select

## The following object is masked from 'package:dplyr':
##
## select

## Loading required package: lattice

##
## Loaded mixOmics 6.8.0
##
## Thank you for using mixOmics! Learn how to apply our methods with our tutorials on www.mixOmics.org, vignette and bookdown on https://github.com/mixOmicsTeam/mixOmics
## Questions: email us at mixomics[at]math.univ-toulouse.fr
## Bugs, Issues? https://github.com/mixOmicsTeam/mixOmics/issues
## Cite us: citation('mixOmics')

##
## Attaching package: 'mixOmics'

## The following object is masked from 'package:purrr':
##
## map

library(mclust)

## Warning: package 'mclust' was built under R version 3.6.1

## Package 'mclust' version 5.4.5
## Type 'citation("mclust")' for citing this R package in publications.

##
## Attaching package: 'mclust'

## The following objects are masked from 'package:mixOmics':
##
## map, unmap

## The following object is masked from 'package:purrr':
##
## map

library(heatmaply)

## Warning: package 'heatmaply' was built under R version 3.6.1

## Loading required package: viridis

## Warning: package 'viridis' was built under R version 3.6.1

## Loading required package: viridisLite

## Registered S3 method overwritten by 'seriation':
## method from
## reorder.hclust gclus

##
## ======================
## Welcome to heatmaply version 0.16.0
##
## Type citation('heatmaply') for how to cite the package.
## Type ?heatmaply for the main documentation.
##
## The github page is: https://github.com/talgalili/heatmaply/
## Please submit your suggestions and bug-reports at: https://github.com/talgalili/heatmaply/issues
## Or contact: <tal.galili@gmail.com>
## ======================

# Datas reading

# Data type ("areas" or "growth" for growth rate)
dname= "growth"
# If Log transformation for areas ("TRUE" if yes or "FALSE" if no)
transflog=TRUE
# Replicate number
numrep=1
# To keep Control cells (TRUE) or not (FALSE)
control=FALSE

if(dname=="areas"){
 if(transflog==T){dataname="Log (areas)"}else{dataname="Areas"}
}else{dataname="Growth Rate"}

Data = read.table(paste("NtdTSL1datas",dname,".csv",sep=""),header=T,sep=";")
if (control==TRUE){
Data=Data[which(Data$replicate==numrep),which(str_detect(colnames(Data),"replicate")==FALSE)]
}else{
Data=Data[which(Data$replicate==numrep & Data$population!="c"),which(str_detect(colnames(Data),"replicate")==FALSE)]
}

colnames(Data)=str_replace(colnames(Data),"population","Sample")
if(dname=="areas"){
 colnames(Data) = str_replace(colnames(Data),"area","")
}else{
 colnames(Data)=c("t1_t2","t2_t3","t3_t4","t4_t5","Sample")
}

if (dname=="areas" & transflog=="TRUE"){
 Data[,1:5]=log(Data[,1:5])
}
rownames(Data)=c(1:nrow(Data))
Tmax=max(which(colnames(Data) !="Sample"))

Here we load Growth Rate for raplicate 1. Dataset chart contains 11171 rows and 5 columns.

By looking at Log(areas), we need to check areas positive growth overtime for every cell. Same for growth rates. Cells that do not respect that constraints will be removed.

if(dname=="areas"){
DDiff=apply(Data[,1:Tmax],1,diff)
Idiff=which(apply(DDiff<0,2,sum)!=0)
}

if (dname=="growth"){
 Idiff=which(apply(Data[,1:Tmax]<0,1,sum)!=0)
}

Data=Data[-Idiff,]

Dataset chart now contains 10674 cells.

rm(DDiff,Idiff)
summary(Data)

## t1_t2 t2_t3 t3_t4 t4_t5
## Min. :0.0002888 Min. :0.002122 Min. :0.0000 Min. :0.0000
## 1st Qu.:0.1035098 1st Qu.:0.186965 1st Qu.:0.2118 1st Qu.:0.1682
## Median :0.1475071 Median :0.244589 Median :0.2572 Median :0.2225
## Mean :0.1527232 Mean :0.230929 Mean :0.2489 Mean :0.2195
## 3rd Qu.:0.2027926 3rd Qu.:0.286969 3rd Qu.:0.2990 3rd Qu.:0.2782
## Max. :0.9693081 Max. :1.133277 Max. :1.1818 Max. :1.0853
## Sample
## c: 0
## m:4760
## p:5914
##
##
##

df=melt(Data,variable.name="Temps")

ggplot(df[which(df$Sample=="m"),], aes(x=value, colour=Temps)) +
 geom_density()+
 ggtitle("Density for Minus")+
 xlim(c(min(df$value),max(df$value)))


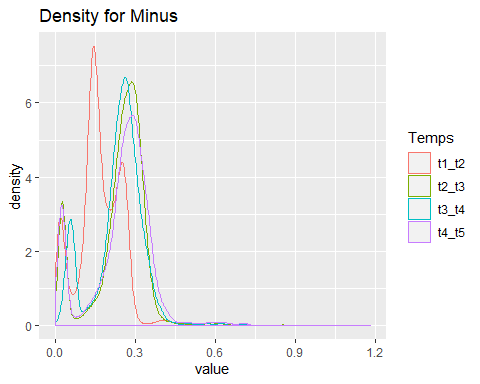


ggplot(df[which(df$Sample=="p"),], aes(x=value, colour=Temps)) +
 geom_density()+
 ggtitle("Density for Plus")+
 xlim(c(min(df$value),max(df$value)))


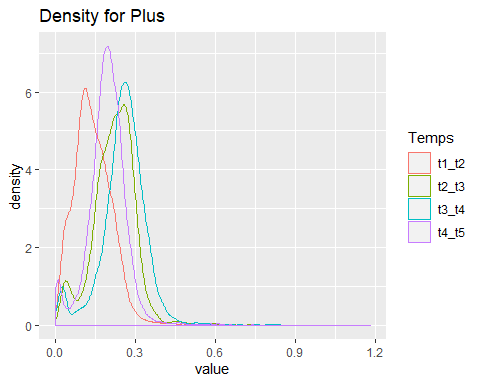


if(control==T){
 ggplot(df[which(df$Sample=="c"),], aes(x=value, colour=Temps)) +
 geom_density()+
 ggtitle("Density for Control")+
 xlim(c(min(df$value),max(df$value)))
}

# Auxiliary fonctions for clustering
###############################################
clustCentroid = function(data,clustering){
 center=matrix(0,nrow=max(clustering),ncol=ncol(data))
 for (k in 1:max(clustering)){
 I=which(clustering==k)
 if (length(I)==1){
 center[k,]=data[I,]
 }else{
 center[k,]=colMeans(data[I,])
 }
 }
 return(center)
}

# Plot of centers
centreClust = function(clustSize, clustCentroids){
 clustMelt = melt(clustCentroids)
 colnames(clustMelt) = c("cluster","temps","value")
 clustMeltMerge = merge(clustMelt, clustSize, by.x = "cluster", by.y = "row.names",all.x=T)
 colnames(clustMeltMerge)[1:3] = c("cluster","temps", "value")
 colnames(clustMeltMerge)[ncol(clustMeltMerge)] = "size"
 clustMeltMerge$cluster = as.factor(clustMeltMerge$cluster)
 g1=ggplot(clustMeltMerge,aes(x=temps,y=value,group=cluster,colour=cluster))+
 geom_line()+
 geom_point()+
 guides(col = guide_legend(nrow = 10))
 return(g1)
}


PlotClustGp <- function(clustering,Ech){
 library(ggplot2)
 classif=as.character(clustering)
 classif[which(nchar(classif)==1)]=paste("0",classif[which(nchar(classif)==1)],sep="")
 classif = paste("Cl-",classif,sep="")
 dd=data.frame(classif=classif,Ech=as.factor(Ech))
 ggplot(dd,aes(x = classif,fill = Ech)) +
 geom_bar(position = "fill")+
 theme(axis.text.x = element_text(angle = 90))+
 ylab("Proportion")+
 xlab("Cluster")
}

PlotMatrixARI = function(ari){
 library(reshape2)
 library(scales)
 library(ggplot2)
 co=melt(ari)
 co$Var1=as.factor(co$Var1)
 co$Var2=as.factor(co$Var2)
 gari = ggplot(co, aes(Var1, Var2)) + # x and y axes => Var1 and Var2
 geom_tile(aes(fill = value)) + # background colours are mapped according to the value column
 #geom_text(aes(fill = co$value, label = round(co$value, 2)),size=5) + # write the values
 scale_fill_gradient2(low = "blue",
 mid = "white",
 high = "red",
 midpoint = 0.5) + # determine the colour
 theme(panel.grid.major.x=element_blank(), #no gridlines
 panel.grid.minor.x=element_blank(),
 panel.grid.major.y=element_blank(),
 panel.grid.minor.y=element_blank(),
 panel.background=element_rect(fill="white"), # background=white
 axis.text.x = element_text(angle=90, hjust = 1,vjust=1,size = 12,face = "bold"),
 plot.title = element_text(size=20,face="bold"),
 axis.text.y = element_text(size = 12,face = "bold")) +
 #ggtitle("ARI Plot") +
 theme(legend.title=element_text(face="bold", size=14)) +
 scale_x_discrete(name="") +
 scale_y_discrete(name="") +
 labs(fill="ARI value")

 #gari=heatmap.2(ari,trace="none",dendrogram="none",scale="none")
 return(gari)
}

ProfilsCluster <-function(Data,clustering){
 A=melt(data.frame(Data,Name=as.factor(rownames(Data)),Clust=as.factor(clustering)))
for (k in 1:max(clustering)){
g=ggplot(A[which(A$Clust==k),],aes(x=variable,y=value,group=Name,colour=Sample))+
 geom_line()+
 geom_point()+
 ggtitle(paste("Cluster",k,sep=""))
print(g)
}
 rm(A)
}

# Calculating N_k*100/N, N_{c,k}*100/N_c et N_{o,k}*100/N_o
PourcEffectSample<-function(Clustering,Sample){
A=cbind(table(Clustering),table(Clustering,Sample))
A=apply(A,2,function(x){round(x*100/sum(x),digits=2)})
colnames(A)[1]=c("Tot")
A=data.frame(Cl=paste("Cl",as.factor(unique(Clustering)),sep=""),A)
return(A)
}

# Graphical proportion representation
PlotPourcEffectSample<-function(A){
ggplot(data=melt(A[,-2],id=1), aes(x=Cl, y=value, fill=variable)) +
geom_bar(stat="identity", color="black", position=position_dodge())+ scale_fill_brewer(palette="Blues")+
 ylab("Pourcent")+
 xlab("Clusters")
}

# Clustering (CAH)

## Ward

hierarchical cluster analysis with euclidian distance and mesure of agglomerates by Ward’s method :

H=hclust(dist(Data[,1:Tmax]),method="ward.D2")

Evolution within-cluster weight function of cluster number :

plot(sort(H$height,decreasing=T)[1:40], ylab="Height",xlab="Nb Cluster", pch=20)
points(sort(H$height,decreasing=T)[1:40],type="l")


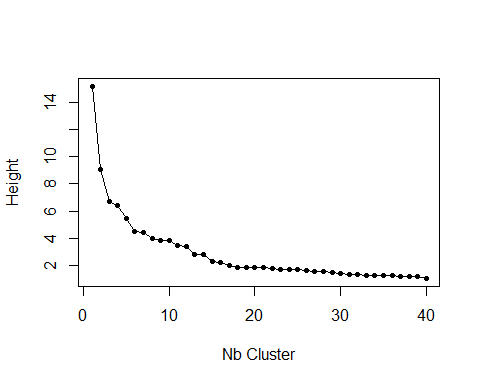


Classifications comparison by ARI :

Kmax=40
Clust=NULL
for (k in 2:Kmax)
 Clust=rbind(Clust,cutree(H,k))

ARI=matrix(1,nrow=Kmax-1,ncol=Kmax-1)
for (j in 1:(Kmax-2)){
 for (i in (j+1):(Kmax-1)){
 ARI[j,i]=ARI[i,j]=adjustedRandIndex(Clust[j,],Clust[i,])
 }
}

rownames(ARI)=colnames(ARI)=seq(2,Kmax,1)
PlotMatrixARI(ARI)


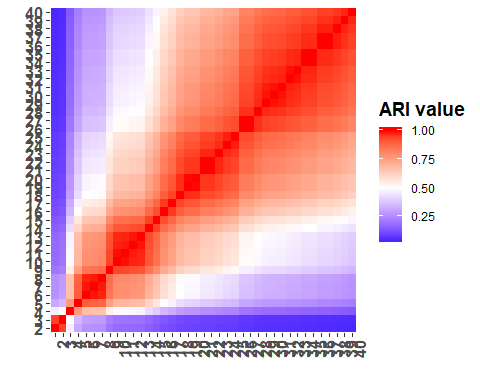


## For 20 Clusters

Profile of cluster centers :

clustSize=table(Clust[19])
clustCentroids=clustCentroid(Data[,1:Tmax],Clust[19,])
ggplotly(centreClust(table(Clust[19]), clustCentroid(Data[,1:Tmax],Clust[19,])))

Clusters headcount :

table(Clust[19,])

##
## 1 2 3 4 5 6 7 8 9 10 11 12 13 14 15
## 67 826 406 1315 788 535 684 392 1106 689 137 803 108 261 553
## 16 17 18 19 20
## 604 903 74 342 81

Sample=factor(Data[,"Sample"],levels=c("m","p"))
Clustering=Clust[19,]
A=PourcEffectSample(Clustering,Sample)
rmarkdown::paged_table(A)

Suppopulation proportion for each cluster :

PlotClustGp(Clust[19,],Data[,"Sample"])


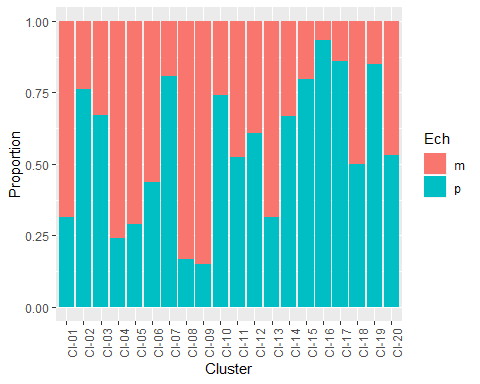


PlotPourcEffectSample(A)


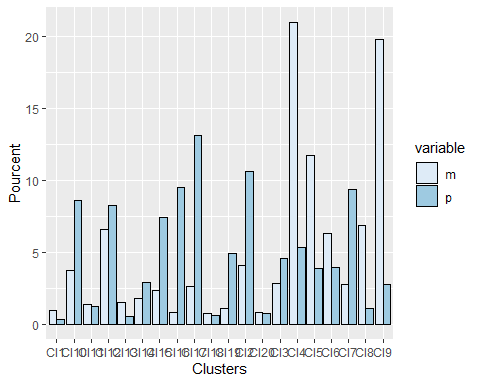


Cells distribution for each cluster :

df=melt(data.frame(Data[,-18],Clust=as.factor(Clust[19,])),variable.name="Temps")

ggplot(df,aes(fill=Temps, y=value, x=Clust)) +
 geom_violin(position="dodge", alpha=0.5)


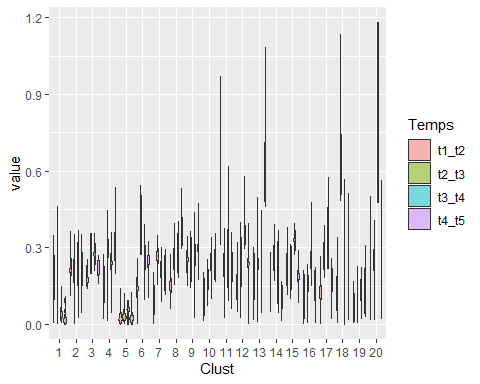

Supplement: Supplementary file 5 [file Data_Sheet_4.ZIP › hierarchical_cluster_analysis/Script_Clustering_GrowthData.docx]
